# Supplementary material for: COZOID: contact zone identifier for visual analysis of protein-protein interactions
Source: BMC Bioinformatics. 2018 Apr 6;19:125. doi: 10.1186/s12859-018-2113-6 (PMC5889581; doi:10.1186/s12859-018-2113-6)
Supplement: Supplementary file 2 — Software build. Executable binary file of the software tool. (ZIP 118,784 kb) [file 12859_2018_2113_MOESM2_ESM.zip › cozoid/java/docs/beansbinding-1.2.1-doc.zip!/org/jdesktop/swingbinding/adapters/JTextComponentAdapterProvider.Adapter.html]

JTextComponentAdapterProvider.Adapter (Beans Binding)


---


|  |  |  |
| --- | --- | --- |
| **Overview** \| **Package** \| **Class** \| **Use** \| **Tree** \| **Deprecated** \| **Index** \| **Help** | |  |
| **PREV CLASS**   NEXT CLASS | **FRAMES**    **NO FRAMES**     **All Classes** |  |
| SUMMARY: NESTED \| FIELD \| CONSTR \| METHOD | DETAIL: FIELD \| CONSTR \| METHOD |  |


---


## org.jdesktop.swingbinding.adapters Class JTextComponentAdapterProvider.Adapter

```
java.lang.Object
  org.jdesktop.swingbinding.adapters.BeanAdapterBase
      org.jdesktop.swingbinding.adapters.JTextComponentAdapterProvider.Adapter
```

**Enclosing class:**: JTextComponentAdapterProvider

---

``` public final class JTextComponentAdapterProvider.Adapter extends BeanAdapterBase ```

---

**Field Summary**

**Fields inherited from class org.jdesktop.swingbinding.adapters.BeanAdapterBase**

`property`


| **Method Summary** | |
| --- | --- |
| `java.lang.String` | `getText_ON_ACTION_OR_FOCUS_LOST()` |
| `java.lang.String` | `getText_ON_FOCUS_LOST()` |
| `java.lang.String` | `getText()` |
| `protected  void` | `listeningStarted()` |
| `protected  void` | `listeningStopped()` |
| `void` | `setText_ON_ACTION_OR_FOCUS_LOST(java.lang.String text)` |
| `void` | `setText_ON_FOCUS_LOST(java.lang.String text)` |
| `void` | `setText(java.lang.String text)` |

**Methods inherited from class org.jdesktop.swingbinding.adapters.BeanAdapterBase**

`addPropertyChangeListener, addPropertyChangeListener, firePropertyChange, getPropertyChangeListeners, getPropertyChangeListeners, isListening, removePropertyChangeListener, removePropertyChangeListener`

**Methods inherited from class java.lang.Object**

`clone, equals, finalize, getClass, hashCode, notify, notifyAll, toString, wait, wait, wait`

**Method Detail**

### getText

```
public java.lang.String getText()
```

---


### getText\_ON\_ACTION\_OR\_FOCUS\_LOST

```
public java.lang.String getText_ON_ACTION_OR_FOCUS_LOST()
```

---


### getText\_ON\_FOCUS\_LOST

```
public java.lang.String getText_ON_FOCUS_LOST()
```

---


### setText

```
public void setText(java.lang.String text)
```

---


### setText\_ON\_ACTION\_OR\_FOCUS\_LOST

```
public void setText_ON_ACTION_OR_FOCUS_LOST(java.lang.String text)
```

---


### setText\_ON\_FOCUS\_LOST

```
public void setText_ON_FOCUS_LOST(java.lang.String text)
```

---


### listeningStarted

```
protected void listeningStarted()
```

:   **Overrides:**: `listeningStarted` in class `BeanAdapterBase`

---


### listeningStopped

```
protected void listeningStopped()
```

:   **Overrides:**: `listeningStopped` in class `BeanAdapterBase`


---


|  |  |  |
| --- | --- | --- |
| **Overview** \| **Package** \| **Class** \| **Use** \| **Tree** \| **Deprecated** \| **Index** \| **Help** | |  |
| **PREV CLASS**   NEXT CLASS | **FRAMES**    **NO FRAMES**     **All Classes** |  |
| SUMMARY: NESTED \| FIELD \| CONSTR \| METHOD | DETAIL: FIELD \| CONSTR \| METHOD |  |


---
